# Supplementary material for: Safety of Anti-Reelin Therapeutic Approaches for Chronic Inflammatory Diseases
Source: Cells. 2024 Mar 27;13(7):583. doi: 10.3390/cells13070583 (PMC11011630; doi:10.3390/cells13070583)
Supplement: Supplementary file 1 [file cells-13-00583-s001.zip › cells-2909077-supplementary.pdf]

Appendix

Safety of Anti-Reelin Therapeutic Approaches for Chronic Inflammatory Diseases

Laurent Calvier, Ph.D.[1,2]*; Anna Alexander[1,2]; Austin T. Marckx[1,2]; Maria Z. Kounnas, Ph.D. [3]; Murat Durakoglugil[1,2]; Joachim Herz, M.D.[1,2,4,5]

[1] Department of Molecular Genetics, University of Texas (UT) Southwestern Medical Center, Dallas, USA.

[2] Center for Translational Neurodegeneration Research, UT Southwestern Medical Center, Dallas, USA.

[3] Reelin Therapeutics Inc., La Jolla CA, USA.

[4] Department of Neuroscience, UT Southwestern Medical Center, Dallas, USA.

[5] Department of Neurology and Neurotherapeutics, UT Southwestern Medical Center, Dallas, USA.

*Corresponding author:

Laurent Calvier, Ph.D.

Molecular Genetics

UT Southwestern Medical Center

5323 Harry Hines Blvd.

Dallas, TX 75390-9046

calvier.laurent@gmail.com

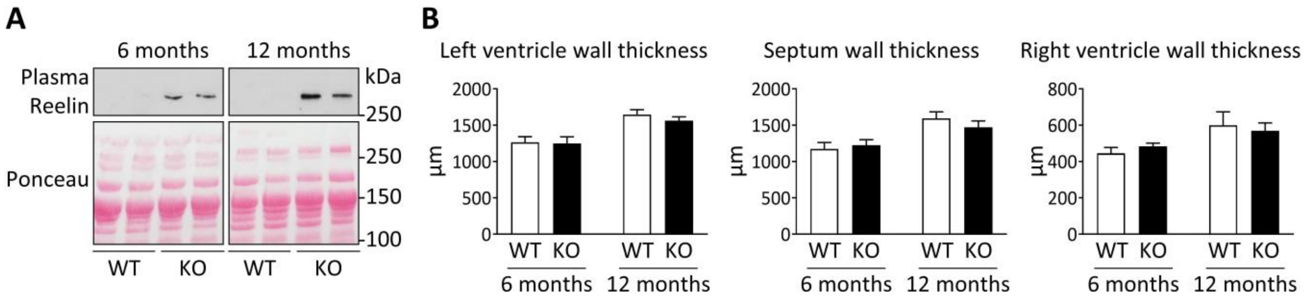

Figure A1. Long-term Reelin depletion has no adverse effect on organ functions.

Reelin conditional KO mice and WT littermates, both on LDLR KO background, were fed a western diet for 4 months (for the 6-month time point) or for 10 months (for the 12-month time point). At the end of each time point, various organs including the heart, kidney, and liver were analyzed to find any adverse effect of prolonged Reelin depletion under the physiological stress imposed by a western diet. (**A**) Reelin protein expression was evaluated in plasma by western blot. (**B**) Cardiac wall thickness was measured on the H&E slides.
